# Charge transfer magnetoexciton formation at vertically coupled quantum dots

**DOI:** 10.1186/1556-276X-7-585

**Published:** 2012-10-23

**Authors:** Willian Gutiérrez, Jairo H Marin, Ilia D Mikhailov

**Affiliations:** 1 Escuela de Física, Universidad Industrial de Santander, A. A. 678, Bucaramanga, Colombia; 2 Escuela de Física, Universidad Nacional de Colombia, A.A. 3840, Medellín, Colombia

**Keywords:** Magnetoexciton, Vertically coupled quantum dots, Giant dipolar momentum, Galerkin method, Density of energy states.

## Abstract

A theoretical investigation is presented on the properties of charge transfer excitons at vertically coupled semiconductor quantum dots in the presence of electric and magnetic fields directed along the growth axis. Such excitons should have two interesting characteristics: an extremely long lifetime and a permanent dipole moment. We show that wave functions and the low-lying energies of charge transfer exciton can be found exactly for a special morphology of quantum dots that provides a parabolic confinement inside the layers. To take into account a difference between confinement potentials of an actual structure and of our exactly solvable model, we use the Galerkin method. The density of energy states is calculated for different InAs/GaAs quantum dots’ dimensions, the separation between layers, and the strength of the electric and magnetic fields. A possibility of a formation of a giant dipolar momentum under external electric field is predicted.

## Background

Over the last decades, small semiconductor systems with discrete energy spectra, known as quantum dots (QDs) and that are analogs of atoms, have fired the imagination of researchers in many fields of physics [1-3]. Unlike in atomic systems, a variety of geometries and configurations of the charge state in these artificial atoms are possible. These particular features make possible to consider QDs as building blocks for the fabrication of more complex structures, such as solid-state artificial molecules, case in which coupled QDs act similar to coupled atoms in a natural molecule [4-6]. Although a diverse range of technologies have been implemented to fabricate QDs, in the case of artificial molecules, there has been a growing interest in spontaneous formation techniques by utilizing self-assembling phenomena on crystal surfaces. One of the most interesting manifestations of this phenomenon is the process of vertical self-alignment of the stacked self-assembled quantum dots (SAQDs) [7-9]. These wonderful structures composed of two or more vertically stacked SAQDs have the advantage of possessing different morphologies such as disks, pyramids, rings or lenses with very few imperfections. Also, they are, in general, thin layers and have, for the most part, a small height-to-base aspect ratio, which is an significant advantage that allows us, on one hand, to modify essentially the energy spectrum of the particles confined within the heterostructure, making them more stable and, on the other hand, to use simple theoretical models.

Currently, there is significant interest in understanding the role of the quantum tunneling of charge carriers between vertically coupled QDs, driven not only by a fundamental nature of this phenomenon but also by their potential applications. Particularly, many efforts have been focused on the theoretical study of the simplest configuration of a QD molecule, namely, a pair of QDs coupled by tunneling [10-13]. In part, interest in such structure arises from its application as a possible gate in a quantum processor required to entangle different states of an electron-hole pair created optically [14-16]. Different exciton states can be disentangled by preventing the tunneling through the application of an electric field along the growth direction. Formed in this way, one of the untangled states, charge-transfer exciton has two important characteristics: an extremely long lifetime and a permanent dipole moment [17,18]. Additionally, its optical properties can be controlled by means of an external magnetic field.

In this work, we consider heterostructures consisting of two vertically aligned hill-shaped InAs/GaAs SAQDs. The QDs’ morphology has been modeled using a special shape which provides an almost parabolic confinement, allowing us to perform a relatively simple calculation of the exciton spectrum. In our model, a two-dot molecule with a single captured electron-hole pair can remain in one of two possible configurations with different dipole moments. In the first case, when the electron and the hole are located at the same dot (on-site exciton), the dipole moment is small, while in the second case, as the particles are situated at different dots (charge transfer exciton), the dipole moment can be very large. In order to illustrate how the electric and magnetic fields applied along to the heterostructure growth direction can facilitate or block a transition between two possible carriers configurations and in this way control electro-optical properties of such structures, we have calculated their densities of states and the averaged values of the dipole moment for different temperatures.

## Methods

### Theoretical model

**Figure 1:**
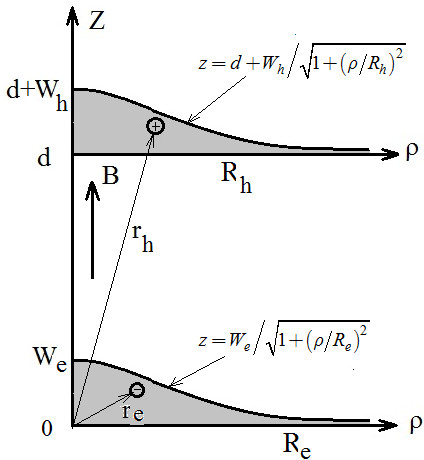
**Schematic illustration of the cross-section profile of vertically coupled quantum dots in the radial direction.** Here ${\vec{r}}_{\text{e}}$ and ${\vec{r}}_{\text{h}}$ are position vectors of the electron and the hole, respectively.

In the case of the charge transfer exciton, we consider a model of two vertically coupled InAs QDs in the form of the axially symmetrical thin layers with the electron located at the lower dot of the radius *R*_e_ and with the thickness at the top *W*_e_ and the hole located at the upper dot with the corresponding parameters *R*_h_ and *W*_h_. In the case of the on-site exciton, both carriers are located at the lower QD. Both layers are considered to be imbedded inside a matrix of the material GaAs. A schematic representation of this system is showed in the Figure 1. Here and in what follows, the variables labeled by indices e and h are referred to the electron and to the hole, respectively. The separation between dots along the *z*-axis is denoted by *d*. In order to obtain results that allows us to analyze qualitatively a transformation of the properties of the structure under external electric and magnetic fields, we adopt a model of the axially symmetrical hill-shaped QD with infinite-barrier confinement and a special shape in which the profile of the dot, given by the dependencies of the thickness of the layers *w*_*p*_, *p* = *e*, *h* on the distance from the axis *ρ*_*p*_, *p* = *e*, *h*, are defined as follows:

(1) $$w_{\text{e}}\left(\rho_{\text{e}}\right)=\frac{W_{\text{e}}}{\sqrt{1+{\left(\rho_{\text{e}}/R_{\text{e}}\right)}^{2}}};\;w_{\text{h}}\left(\rho_{\text{h}}\right)\frac{W_{\text{h}}}{\sqrt{1+{\left(\rho_{\text{h}}/R_{\text{h}}\right)}^{2}}}$$

As we show below, such profile provides in-plane parabolic confinement for which the mathematical treatment is significantly easier. For the same reason, we adopt a model with infinite barrier confinement, assuming that dielectric mismatch is much smaller than the mismatches of the conduction and the valence bands; therefore, the probability for the self-tunneling of the particles between QDs is depreciable in comparison with the tunneling provided by the external electric field. In this way, we assume that confinement potential for the electron and for the hole is equal to zero inside the QDs and to infinity otherwise, defined in cylindrical coordinates as:

(2) $$\begin{array}{l} V_{e}\left(r_{e}\right)=\left\{\begin{array}{c} 0\;if\;0<z_{e}<w_{e}\left(\rho_{e}\right)\\ \infty \;otherwise \end{array}\right)\;,\\ V_{h}\left(r_{h}\right)=\left\{\begin{array}{c} 0\;if\;d<z_{h}<d+w_{h}\left(\rho_{h}\right)\\ \infty \;otherwise \end{array}\right)\;\text{,} \end{array}$$

Here, three-dimensional position vectors of the electron and the hole in the cylindrical coordinates are **r**_*p*_ = (*ρ*_*p*_, *ϑ*_*p*_, *z*_*p*_); *p* = *e*, *h*.

The values of the physical parameters pertaining to InAs used in our calculations are dielectric constant *ε* = 15.2, the effective masses in the InAs material layer for the electron *m*_e_ = 0.04*m*_0_ and for hole *m*_h_ = 0.34*m*_0_, the conduction and the valence bands offsets in junctions are *V*_0e_ = 450 meV and *V*_0h_ = 316 meV, respectively [19].

As the quantum dots and the exciton sizes under consideration are much larger than the unit cell of the material, the effective-mass approximation is a suitable approach; therefore the resulting model Hamiltonian of the electron-hole pair in the presence of uniform magnetic and electric fields oriented along the *z*-axis, perpendicular to the plane of QDs, can be written as:

(3) $$H=\sum_{p=e,h}\left[\frac{1}{2m_{\text{p}}}{\left(p_{p}-\frac{q_{\text{p}}}{c}A_{p}\right)}^{2}+V_{p}\left(r_{p}\right)\mp q_{p}Fz_{p}\right]-\frac{e^{2}}{\varepsilon r_{\text{eh}}}\text{;}$$

Here, $r_{\text{eh}}=\sqrt{{\left(z_{e}-z_{h}\right)}^{2}+r_{e}^{2}+r_{h}^{2}-2r_{e}r_{h}\cos\left(\vartheta_{e}-\vartheta_{h}\right)}$ is the electron-hole separation; *m*_p_ and **p**_p_ are the effective masses and the momentum vectors of the particles, respectively; and the parameter *q*_p_ = ± *e* gives their charges. Choosing the gauge for electron and hole vector potentials as **A**_*e*_ = (**B** × **r**_*e*_) / 2; **A**_*h*_ = (**B** × **r**_*h*_)/2, where **B** is the magnetic field (assumed to be uniform here), the Hamiltonian (3) can be reduced to the following dimensionless form:

(4) $$\begin{array}{l} H=H_{0\text{e}}+H_{0\text{h}}-2/r_{\text{eh}};\\ H_{0p}=-\eta_{p}\left[-\Delta_{p}+\gamma^{2}\rho_{p}^{2}/4-i\gamma s_{p}\partial /\partial \vartheta_{p}\mp \alpha \;z_{p}\right]+V_{p}\left(r_{p}\right);\\ p=e,h;\eta_{\text{h}}=\mu /m_{\text{h}};\;\eta_{\text{e}}=\mu /m_{\text{e}};\;s_{\text{h}}=+1;\;s_{\text{e}}=-1 \end{array}$$

In these equations *H*_0e_ and *H*_0h_ represent the Hamiltonians of the unbound electron and hole respectively, confined inside their heterostructures. The following units are used in the dimensionless Hamiltonian (4), the exciton effective Bohr radius *a*_0_^∗^ = *ℏ*^2^*ε*/*μ e*^2^ as the unit of length, the effective Rydberg *Ry* * = *e*^2^/2*ε a*_0_^∗^ = *ℏ*^2^/2*μ a*_0_^∗^^2^ as the energy unit, and *γ* = *eℏB*/2*μ c Ry* * and *α* = *ea*_0_^*^F/*Ry* * as the units of the magnetic and electric field strengths respectively, with *μ* = *m*_e_*m*_h_/(*m*_e_ + *m*_h_) being the reduced mass. The parameters *η*_e_, *η*_h_ satisfy the relation *η*_e_ + *η*_h_ = 1; in our calculations, we assume that *η*_e_ > *η*_h_.

Taking into account that typically, in actual self-assembled QDs, the height is much smaller than the lateral dimensions; one can take an advantage of the adiabatic approximation, which allows us to exclude temporarily the rapid particle motions along *z*-axis from consideration and, in this way, to reduce the dimensionality of the initial three-dimensional problem. Following the adiabatic procedure, described in reference [12], one can obtain the two-dimensional effective Hamiltonian, which describes only the in-plane particle motion of the form:

(5) $$\begin{array}{l} H^{\left(2D\right)}=H_{0\text{e}}^{\left(2D\right)}+H_{0\text{h}}^{\left(2D\right)}-\frac{2}{\sqrt{d{\;}^{2}+{\left|\rho_{\text{e}}-\rho_{\text{h}}\right|}^{2}}};\\ H_{0p}^{\left(2D\right)}=-\eta_{p}\left[-\Delta_{p}^{\left(2D\right)}+\frac{\gamma^{2}\rho_{p}^{2}}{4}-i\gamma s_{p}\frac{\partial }{\partial \vartheta_{p}}+\frac{\pi^{2}}{w_{p}^{2}\left(\rho_{p}\right)}\mp \alpha 〈z_{p}〉\right]\;;\\ p=e,h \end{array}$$

Here, 〈*z*_*p*_〉,  *p* = *e*, *h* are the mean values of z-coordinate of the corresponding particle. Finally, for the selected profile (1) the Hamiltonian (5) describes two particles in 2D quantum dot with parabolic confinement:

(6) $$\begin{array}{l} H^{\left(2D\right)}=E_{0}-\sum_{p=e,h}\eta_{p}\left[-\Delta_{p}^{\left(2D\right)}+i\gamma s_{p}\frac{\partial }{\partial \vartheta_{p}}+\frac{\omega_{p}^{2}\rho_{p}^{2}}{4}\mp \alpha 〈z_{p}〉+\frac{\pi^{2}}{W_{p}^{2}}\right]\\ \;-\frac{2}{\sqrt{d{\;}^{2}+{\left|\rho_{\text{e}}-\rho_{\text{h}}\right|}^{2}}};\omega_{p}^{2}=\frac{4\pi^{2}}{W_{p}^{2}R_{p}^{2}}+\gamma^{2} \end{array}$$

Finding the eigenfunctions of the Hamiltonian (6) allows us to introduce relative and center of mass coordinates **r** = **r**_e_ − **r**_h_ and **R** = *η*_h_**r**_e_ + *η*_e_**r**_h_, respectively. Thus, the Hamiltonian (6) is reduced to:

(7) $$\begin{array}{l} H=E_{0}+H_{R}+H_{r}+U;\\ E_{0}=\sum_{p=e,h}\eta_{e}\pi^{2}/W_{p}^{2};\;\\ H_{R}=-\eta_{e}\eta_{h}\Delta_{R}^{\left(2D\right)}+\left(\eta_{e}\omega_{e}^{2}+\eta_{h}\omega_{h}^{2}\right)R^{2}/4;\\ H_{r}=-\Delta_{r}^{\left(2D\right)}+i\gamma \frac{\partial }{\partial \vartheta }+\frac{1}{4}\left(\eta_{e}^{3}\omega_{e}^{2}+\eta_{h}^{3}\omega_{h}^{2}\right)r^{2}-\frac{2}{\sqrt{d{\;}^{2}+r^{2}}};\;\\ U=\frac{1}{2}\left(\eta_{e}^{2}\omega_{e}^{2}-\eta_{h}^{2}\omega_{h}^{2}\right)R\cdot r \end{array}$$

Here, *E*_0_ is the background energy; second and third terms *H*_*R*_ and *H*_*r*_ describe the center of mass and relative motions, respectively, and they correspond to the Hamiltonians for two independent central force problems; while the last term *U* presents a perturbation. The Hamiltonian *H*_*R*_ coincides with one of the circular oscillator, whose eigenvalues can be found exactly and its eigenfunctions can be expressed in terms of the generalized Laguerre polynomials. It is seen that the Hamiltonian (7) becomes completely separable for a particular case when the external magnetic field is zero (*γ* = 0), and the geometric parameters of QDs satisfy the following condition:

(8) $$\eta_{\text{e}}\omega_{\text{e}}=\eta_{\text{h}}\omega_{\text{h}}\text{.}$$

In this case, *U* = 0, and eigenvalues of the Hamiltonian (7) are a sum of the background energy *E*_0_, the center of mass energy *E*_R_ defined by two quantum numbers, radial *N* and angular *M*, and the relative energy *E*_r_ depending on four quantum numbers, radial *n* and angular *m* (*N*, *n* = 0, 1, …; *M*, *m* = 0, ± 1, …):

(9) $$\begin{array}{l} E=E_{0}+E_{\text{R}}\left(N,M\right)+E_{\text{r}}\left(n,m\right);\\ E_{\text{R}}\left(N,M\right)=\left(\left|M\right|+2N+1\right)\sqrt{\eta_{\text{e}}\eta_{\text{h}}\left(\eta_{\text{e}}\omega_{\text{e}}^{2}+\eta_{\text{h}}\omega_{\text{h}}^{2}\right)\;} \end{array}$$

The corresponding wave functions are:

(10) $$\begin{array}{l} \Psi_{N,M,n,m}^{\left(0\right)}\left(R,\Theta ,r,\vartheta \right)=Ce^{\mathrm{iM\Theta}}e^{\mathrm{im\vartheta}}R^{\left|M\right|}e^{-\Lambda R^{2}/2}L_{N}^{\left(M\right)}\left(\Lambda R^{2}\right)\Phi_{n,m}\left(r\right);\\ \;\Lambda =\frac{1}{2}\sqrt{\left(\eta_{e}\omega_{e}^{2}+\eta_{h}\omega_{h}^{2}\right)/\eta_{e}\eta_{h}}\text{,} \end{array}$$

where *L*_*N*_^(*M*)^(*x*) are generalized Laguerre polynomials and *Φ*_*n*,*m*_(*r*) is the radial part of the wave function describing the relative coordinates evolution, which is a solution of the following ordinary differential equation:

(11) $$\begin{array}{l} -\frac{1}{r}\frac{d}{dr}r\frac{d\Phi_{n,m}\left(r\right)}{dr}\\ \;+\left[\frac{m^{2}}{r^{2}}+\gamma m+\frac{1}{4}\left(\eta_{e}^{3}\omega_{e}^{2}+\eta_{h}^{3}\omega_{h}^{2}\right)r^{2}-\frac{2}{\sqrt{d{\;}^{2}+r^{2}}}\right]\Phi_{n,m}\left(r\right)\\ \;=E_{r}\left(n,m\right)\Phi_{n,m}\left(r\right)\text{.} \end{array}$$

Once Equation (11) is solved and the set of wave functions (10) is found then it can be used as the basis to calculate the energy corrections due to the presence of the perturbation *U* in the Hamiltonian (7) in the framework of the so-called exact diagonalization or Galerkin method.

## Results and discussion

In our numerical work, we solve Equation (11) by using the trigonometric sweep method [20] initially for *d* = 0 and later for *d* ≠ 0. The eigenfunctions and eigenvalues found in the first calculation were used for calculating the energy levels *E*_*k*_^(0)^, *k* = 1, 2, 3… of the *onsite exciton* (the electron and the hole are mainly situated at the same QD), while the results of the second calculation were used to find the energy levels *E*_*k*_^(*t*)^, *k* = 1, 2, 3… of the *charge transfer exciton* (the electron and the hole are mainly situated at different QDs).

Once the energies of the two possible exciton configurations *E*_*k*_^(0)^ and *E*_*k*_^(*t*)^, *k* = 1, 2, 3 … are calculated, then corresponding curves of the density of energy states *ρ*^(0)^(*E*) and *ρ*^(*t*)^(*E*) can be found by using the following relations:

(12) $$\begin{array}{l} \rho^{\left(0\right)}\left(E\right)=\sum_{k}f\left(E-E_{k}^{\left(0\right)}\right)\;;\\ \rho^{\left(t\right)}\left(E\right)=\sum_{k}f\left(E-E_{k}^{\left(t\right)}\right)\;;\\ f\left(x\right)=\exp\left(-x^{2}/2s^{2}\right)/s\sqrt{2\pi }\text{.} \end{array}$$

**Figure 2:**
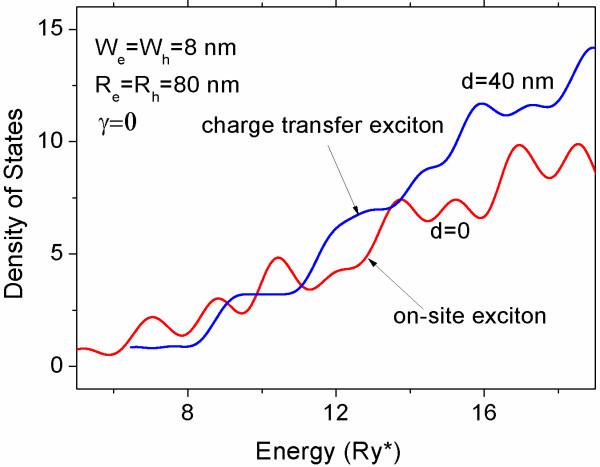
**Density of states for exciton confined in vertically coupled QDs.** The red line corresponds to on site exciton, and the blue line corresponds to charge transfer exciton.

Here, the parameter *s* is a natural width of the individual spectral line of the Gaussian shape. In Figure 2, we present an example of calculations performed for densities of state of the on-site and charge transfer excitons confined in vertically coupled QDs of radii *R*_e_ = *R*_h_ = 80 nm, of height *W*_e_ = *W*_h_ = 8 nm and with separation between them *d* = 40 nm. It is seen that left-side energy threshold of the curve for on-site exciton is lower than for the charge transfer exciton. It is due to the fact that the attraction energy between the electron and the hole in the case when the particles are located at the same QD is higher.

As for the on-site exciton, the projection of the dipole moment over symmetry axis is zero; the external electric field does not change nor its energy levels nor its density of state *ρ*^(0)^(*E*). On the other hand, the energies of the on-site exciton are lower than those of charge transfer exciton; therefore, the dipole moment of this structure with captured exciton in the ground states is almost zero. However, the charge distribution for the ground state can be changed drastically under external electric field *F* applied along the symmetry axis which can provide a reordering of the energy levels due to a lowering of the energy of charge transfer exciton in a value about *eFd*. To verify a validity of this affirmation, we calculate the averaged dipole momentum 〈*p*〉 of the vertically coupled QDs with a single captured exciton at the temperature *T* by using the following relation:

(13) $$〈p〉=\frac{ed\int \rho^{\left(t\right)}\left(E-eFd\right)\;\text{exp}\left(-E/kT\right)dE}{\int \left[\rho^{\left(t\right)}\left(E-eFd\right)+\rho^{\left(0\right)}\left(E\right)\right]\;\text{exp}\left(-E/kT\right)dE}$$

**Figure 3:**
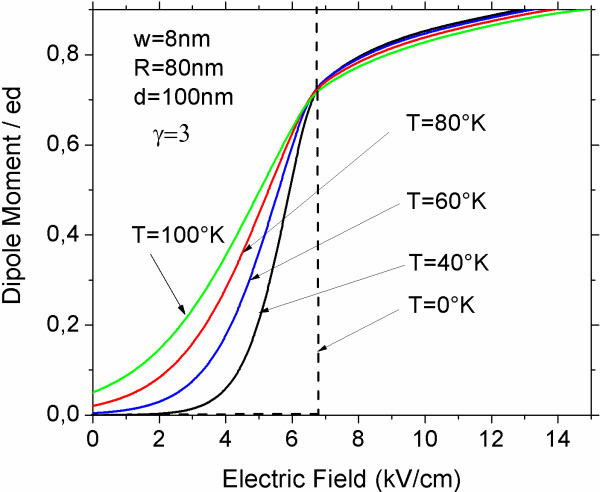
**Electric dipole moment of exciton induced by external electric field.** Dependence of the dipole moment of the exciton captured by vertically coupled QDs on the strength of the external electric field applied along the symmetry axis.

In Figure 3, we present the calculation results of the dipole moment as function of the electric field strength for four different temperatures for the exciton captured by the coupled QDs of radii 50 nm, thickness 4 nm, and the separation between them 40 nm in the presence of the magnetic field *γ* = 3. It is seen for very low temperatures that the dipole moment increases drastically as the external electric field increases and achieves a critical value about *F*_c_ ≈ 7 kV/cm. It is due to the fact that under increasing external electric field, all energy levels of the charge transfer exciton descend, displacing the threshold of the density of states toward the correspondent value of on-site exciton. When electric field reaches a critical value, the positions of two thresholds are interchanged, while the ground state of the exciton suffers a transformation from a configuration corresponding to on-site exciton up to a configuration of the charge transfer exciton. Such transformation is accompanied by a drastic growth of the dipole moment. In our calculations, we find that the gap ∆*E* between thresholds of the densities of states of both exciton types is related to the critical value of the electric field *F*_c_ as *ΔE* = *eF*_c_*d*. In this way, values of both *F*_c_ and ∆*E* are defined by the competition between the structural confinement and the electron-hole interaction.

One can see other peculiarity in Figure 3, the curves for different temperatures all have intersections at the same point. In other words, when the electric field *F* becomes equal to a critical value *F*_c_, the value of the dipole moment is the same for different temperatures. One can explain this result, taking into account that densities of states both for the on-site exciton and for the charge transfer exciton close to their thresholds are almost linear. Therefore, when *F* = *F*_c_, the densities of states of two types of excitons satisfy the relation *ρ*^(*t*)^(*E* − *eF*_c_*d*) = *αρ*^(0)^(*E*). Here, *α* is the ratio of the slopes of the linear parts of curves close to their thresholds. Substituting this relation to Equation (13), one can obtain 〈*p*〉 = *edα* / (1 + *α*), i.e., dipole moment does not depend on temperature.

**Figure 4:**
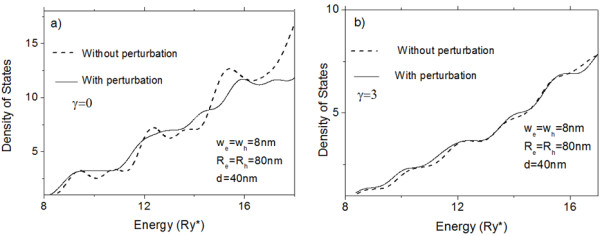
**Density of states corresponding to charge transfer excitons.** The excitons are confined in vertically coupled QDs for two different values of the magnetic field (**a**, **b**). The dashed line corresponds to the case without perturbation and the solid line corresponds to the case with corrections given by the perturbation.

An additional possibility to control the properties of the structure with captured exciton offers the application of the external magnetic field. As the diamagnetic confinement for the charge transfer exciton is stronger than for the on-site exciton, the gap between their ground states energies is changed slightly under external magnetic field, displacing their energies thresholds in different degrees. But more notable is the change which suffers the slope of the curve of the density of states of the charge transfer exciton under external magnetic field. It is clearly seen from Figure 4 where we present densities of states calculated for charge transfer exciton for two different values of the magnetic field.

## Conclusions

In conclusion, our results demonstrate that electro-optical properties of an exciton confined in two vertically coupled quantum dots can be changed remarkably by electric and magnetic fields applied along the growth direction. Especially, we find that through the electric field, a strong dipole moment can be induced; this is due to the tunneling of charge carriers across the potential barrier between dots, which leads to a charge redistribution of electron-hole pair in the structure, passing from a configuration: on-site exciton (the electron and the hole are mainly situated at the same QD) to a configuration charge transfer exciton (the electron and the hole are mainly situated at different QDs).

## Abbreviations

QDs: quantum dots; SAQDs: self-assembled quantum dots.

## Competing interests

The authors declare that they have no competing interests.

## Authors’ contributions

WG and JM carried out the numerical calculations and drafted the manuscript. IDM analyzed and interpreted results, and gave the final approval of the version to be published. All authors read and approved the final manuscript.

## Authors’ information

IDM received the Ph.D. degree from the Physical Technical Institute from Moscow and the D. Sc. degree from the same institute. Currently, IDM is a titular professor at the School of Physics, Universidad Industrial de Santander, Bucaramanga, Colombia. JM received the Ph.D. degree from the Universidad Industrial de Santander; currently, he is an associate professor at the School of Physics, Universidad Nacional de Colombia, Medellín, Colombia.

WG received the Ph.D. degree from the Universidad Industrial de Santander; currently, he is an assistant professor at the School of Physics, Universidad Industrial de Santander, Bucaramanga, Colombia.
